# Unlocking the Potential of *Citrus medica* L.: Antioxidant Capacity and Phenolic Profile across Peel, Pulp, and Seeds

**DOI:** 10.3390/molecules29153533

**Published:** 2024-07-27

**Authors:** Ana Rita Soares Mateus, João David Teixeira, Sílvia Cruz Barros, Carina Almeida, Sónia Silva, Ana Sanches-Silva

**Affiliations:** 1 INIAV—National Institute for Agricultural and Veterinary Research, I.P., Rua dos Lagidos, Lugar da Madalena, Vairão, 4485-655 Vila do Conde, Portugal; anarita.mateus@iniav.pt (A.R.S.M.); david.teixeira@iniav.pt (J.D.T.); silvia.barros@iniav.pt (S.C.B.); carina.almeida@iniav.pt (C.A.); sonia.silva@iniav.pt (S.S.); 2 University of Coimbra, Faculty of Pharmacy, Polo III, Azinhaga de Stª Comba, 3000-548 Coimbra, Portugal; 3 LAQV, REQUIMTE, Laboratory of Bromatology and Pharmacognosy, Faculty of Pharmacy, University of Coimbra, Polo III, Azinhaga de Stª Comba, 3000-548 Coimbra, Portugal; 4 CECA—Centre for Animal Science Studies, ICETA, 4099-002 Porto, Portugal; 5 University of Minho, Department of Chemistry, Largo do Paço, 4704-553 Braga, Portugal; 6 LEPABE—Laboratory for Process Engineering, Environment, Biotechnology and Energy, Faculty of Engineering, University of Porto, Rua Dr. Roberto Frias, 4200-465 Porto, Portugal; 7 AliCE—Associate Laboratory in Chemical Engineering, Faculty of Engineering, University of Porto, Rua Dr. Roberto Frias, 4200-465 Porto, Portugal; 8 Centre of Biological Engineering, 4710-057 Braga, Portugal; 9 LABBELS—Associate Laboratory, 4710-057 Braga, Portugal; 10 Al4AnimalS—Associate Laboratory for Animal and Veterinary Sciences, 1300-477 Lisbon, Portugal

**Keywords:** antioxidants, citron, by-products, phenolic compounds

## Abstract

*Citrus medica* L. is a traditional citrus fruit that is rich in bioactive compounds and has the potential to be used as a natural source of food additives. This study aims to evaluate the antioxidant capacity and characterize the phenolic compounds present in the peels (including flavedo and albedo), pulp, and seeds of citron. The results showed that, compared to the other parts, the pulp had a substantially higher Antioxidant Activity Coefficient (AAC) of 168.2. The albedo and the seeds had significantly lower AAC values, while the green and yellow flavedo showed noteworthy results. *O*-coumaric acid was the predominant phenolic acid in all of the citron fractions; it was found in the highest concentration in albedo (37.54 µg/g FW). Flavanones and flavanols were the primary flavonoids in the pulp, peel, and seeds, with total flavonoid concentration ranging from ~9 µg/g FW in seeds to 508 µg/g FW in the pulp. This research offers significant insights into the antioxidant properties of this ancient fruit, emphasizing its potential applications as a natural source of antioxidants to be used in different applications.

## 1. Introduction

**Figure 1:**
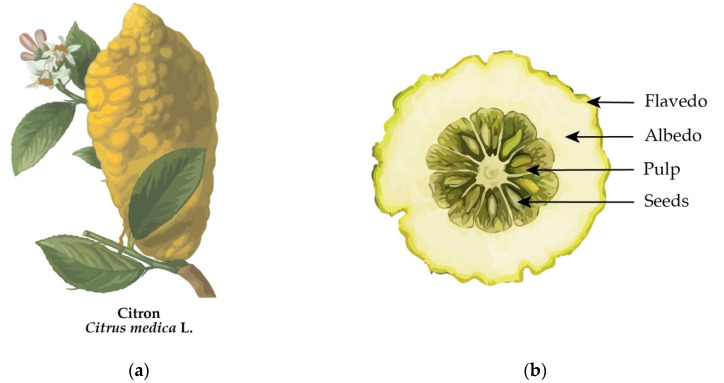
Citron (*Citrus medica* L.): (**a**) representation of whole mature fruit and (**b**) vertical cross-section of citron fruit.

Citron (*Citrus medica* L.) is a large aromatic yellow citrus fruit that resembles a lemon (Figure 1a). Citron is considered an ancient species of the genus *Citrus*, belonging to the Rutaceae family, and the original lemon from which many different cultivars have been developed [1]. *Citrus medica* L. is also called “cedar”, and other denominations like “*fingered citron*” are reported in the literature [2].

One of the principal characteristics of citron is that the peel comprises more than 70% of the fruit’s mass, while the pulp (or endocarp) and seeds represent a minor part of the weight of the fruit. (Figure 1b) [2]. Citron peel, or epicarp, like other citrus fruits, is divided into flavedo and albedo [3]. Albedo, or mesocarp, is the white spongy cellulosic tissue of citron peel, rich in pectin, and represents the major portion of the citron peel. Flavedo, or exocarp, is the pigmented peripheral region with oil glands, rich in essential oils.

Citron is very popular for its medicinal properties. Traditionally, citron is used as medicine for colds, coughs, gastrointestinal disorders, and analgesics [2,4]. Several studies reported its anti-inflammatory [5] and antidiabetic [6,7] properties as well as cytotoxic activity [8].

Despite its benefits, the use of citron in the food industry is very limited. Citron peel is consumed candied with sugar or used to produce a non-alcoholic drink called “*cedrata*”, popular in Italy [9,10]. Most of the produced citrons are processed by the pharmaceutical and cosmetic industries for recovering essential oils due to their citrus aroma and the presence of limonene [11]. 

Nevertheless, citron peel is rich in bioactive compounds and could have more applications in the food industry to reduce food waste. For instance, citron peel extract was used as an antioxidant to stabilize sunflower oil, which effectively prevented the oil oxidation in storage at 65 °C for 5 days [12]. Previously, citron peel essential oil was used as an antimicrobial agent in ready-to-eat salads and effectively inhibited spoilage and pathogenic microorganisms, such as *Listeria monocytogenes* and *Escherichia coli* [13], and its antimicrobial activity has been reported [14,15,16]. Due to the high percentage of peel and its richness of phenolic compounds, citron peel shows great potential to be used as a natural source of food additives. 

This paper aims to carry out a comprehensive study of citron (*Citrus medica* L.), including the evaluation of the antioxidant capacity, through a direct and an indirect method, the determination of total phenolic and total flavonoid content, and also the profile in individual phenolic compounds through UHPLC coupled with high-resolution mass spectrometry (UHPLC-ToF-MS). In view of the total use of this crop, different parts of the fruits were compared, including the peels (including flavedo and albedo), pulp, and seeds of citron, aiming to valorize this traditional citrus fruit.

## 2. Results and Discussion

### 2.1. Antioxidant Capacity Assays

The antioxidant potential of the *Citrus medica* L. fruit parts was assessed using the β-carotene bleaching assay and the DPPH radical scavenging assay. The inhibition percentage (IP %) and AAC obtained by each fruit portion are displayed in Table 1.

The results show that amongst the different portions of the fruit, the Antioxidant Activity Coefficient (AAC) was significantly higher in the pulp (168.2) than in the other portions. While the green and yellow flavedo showed substantial AAC values (117.9 and 111.3, respectively), the albedo and the seeds displayed a much lower value. These results are congruent with the ones published by Menichini et al. [17], in which the authors state that the AAC is higher in the mesocarp (pulp) than in the endocarp (seeds). However, the results seem to be much lower than ours, both in the immature and in the mature fruit cases, because the highest seed AAC from the authors is 7.1, compared to our 19.87. The same applies to the mesocarp. 

The profile of the results of the DPPH scavenging assay is similar to the profile displayed previously in the β-carotene bleaching assay, with the pulp representing the portion where the inhibition percentage is higher (18.38%), followed by the flavedo, although in this case, the yellow flavedo has shown a significantly higher percentage than the green flavedo. Also, the inhibition percentage in the albedo (3.81%) is slightly lower than the inhibition percentage in the seeds (5.46%), contrary to what was registered in the β-carotene bleaching assay.

### 2.2. Phenolic Characteristics Assays

The content of the compounds that contribute to the antioxidant properties was assessed in the other two assays—the TPC and TFC assays. The assessment of both these classes of compounds is compiled in Table 2.

Regarding the TPC, the flavedo presents the highest content of phenolics, although significant differences (*p* < 0.05) between yellow and green portions were observed. The albedo portion had the lowest phenolic content, with 447.1 μg gallic acid equivalents (GAE)/g of fresh fruit (FW). The results show that the portion with the most antioxidant capacity is not the same as the portion with the highest phenolic content, which may be due to the presence of other compounds that provide antioxidant capacity to the fruit, such as vitamins (primarily C but also A, B, and E), anthocyanins, and even carotenoids [18]. 

In a recent paper that studied the TPC in different citrus varieties, Hasan et al. [19] reported TPC values for the juice of Citrus medica of 6–7 mg GAE/100 g. This represents a much lower concentration than the one we observed for the pulp (i.e., the portion from which the juice is extracted) than if we convert to the authors’ choice of concentration unit, which is 56.45 mg GAE/100 g. However, Menichini et al. [17] reported TPC values of 123.1 and 109.4 mg of chlorogenic acid equivalents (CAE)/100 g FW of mesocarp and endocarp, respectively, in Citrus medica portions. These results are much more aligned with ours (56.45 and 71.36 mg GAE/100 g), but even so, they differ in the portion in which the content is higher. Fratianni et al. [9], on the other hand, report that the peel (flavedo) of the Citrus medica variety has 1002.3 μg GAE/g FW of TPC, and the pulp registers 242.73 μg GAE/g FW, showing better agreement with our results. 

Pulp was the portion with the highest TFC (432.9 μg Epicatechin equivalents (EE)/g of fruit), followed by the albedo (339.3 μg EE/g FW), the flavedo (149.1 and 146.2 μg EE/g FW, yellow and green, respectively), and the seeds, which presented the lowest TFC (26.18 μg EE/g FW). Concerning these compounds in *Citrus medica*, Menichini et al. [17] revealed that the mesocarp showed a TFC of 43.1 mg quercetin equivalents (QE)/100 g, which is almost identical to our 43.3 mg EE/100 g in the pulp. The authors also reported a TFC of 37.5 mg QE/100 g in the endocarp, registering a much higher concentration of flavonoids than we were able to in the seeds (2.618 mg EE/100 g).

### 2.3. Determination of Individual Phenolic Compounds

#### 2.3.1. Phenolic Acids

The quantification of 28 individual phenolic compounds was performed with UHPLC-ToF–MS, using a previously validated methodology (Table S1). The results showed that out of 14 phenolic acids, four hydroxycinnamic acid derivates were quantified in the pulp, peel, and seeds of citron, and the total phenolic acid content varied as follows: albedo (37.54 µg/g) > green flavedo (33.96 µg/g) > yellow flavedo (20.24 µg/g) > pulp (13.74 µg/g) > seeds (1.81 µg/g), as shown in Table 3. However, Dadwal et al. [20] found that seeds were the citron fraction with the highest phenolic acid content, rich in chlorogenic and caffeic acids (210.40 µg/g and 155.87 µg/g, respectively), followed by albedo, flavedo, and pulp, where *p*-coumaric acid, *trans*-ferulic acid, gallic acid, chlorogenic acid, caffeic acid, and *trans*-cinnamic acid were identified. Moreover, Fratianni et al. [9] found that the pulp was richer in phenolic acids, where ferulic acid (113.91 µg/g) and gallic acid (22.16 µg/g) were the main phenolic acids quantified. In the peel, gallic acid was the only phenolic acid identified (39.02 µg/g) [9].

**Figure 2:**
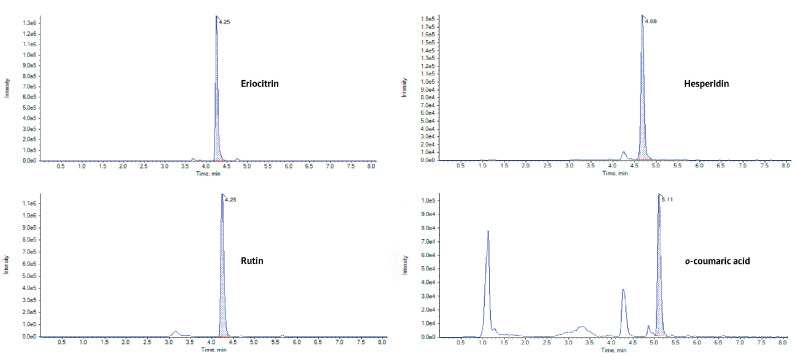
Chromatograms (intensity vs. time (min)) of the four major compounds in the citron pulp sample: eriocitrin (rt = 4.25 min), hesperidin (rt = 4.68 min), rutin (rt = 4.25 min), and o-coumaric acid (rt = 5.11 min), obtained with MultiQuant™ 3.0 software.

In all citron fractions, *o*-coumaric acid was the main phenolic acid, with the highest amount in albedo (37.54 µg/g FW) and the lowest in seeds (0.89 µg/g FW). Albedo was the richest fraction of citron and included higher concentrations of *o*-coumaric acid, the only phenolic acid quantified. Both *o*-coumaric acid and *p*-coumaric acid were quantified in the pulp, flavedo, and seeds. The concentration of *o*-coumaric acid is higher than *p*-coumaric acid in all citrus fractions. The only exception was in seeds, where similar amounts of *p*-coumaric acid and *o*-coumaric acid were found. These results were in agreement with those observed in a recent study on citron where *p*-coumaric acid was determined in all citrus fractions with high amounts in flavedo (28.09 µg/g) and albedo (21.46 µg/g) and lower amounts in pulp (3.90 µg/g) and seeds (3.99 µg/g) [20]. The chromatogram of *o*-coumaric acid in the pulp is represented in Figure 2. 

The highest number of phenolic acids was determined in yellow flavedo, where *o*-coumaric acid had high amounts (9.52 µg/g FW) and *trans*-ferulic acid showed lower levels (1.28 µg/g FW). Chlorogenic acid was only determined in yellow flavedo at 5.54 µg/g FW. The content of chlorogenic acid was 20-fold lower than those described by Dadwal et al. [20] for citron flavedo (109.85 µg/g). Although the presence of chlorogenic acid in yellow flavedo and their absence in green flavedo, comparing total phenolic acid content in green and yellow flavedo, we could conclude that the maturity process leads to a decrease in the phenolic acid content. 

#### 2.3.2. Flavonoids

The results showed that, out of fourteen flavonoids, nine compounds were identified, as shown in Table 4. Flavanones and flavanols were the main flavonoid classes in the pulp, peel, and seeds of citron, and the total flavonoid content varied as follows: pulp (507.58 µg/g) > albedo (169.40 µg/g) > yellow flavedo (85.29 µg/g) > green flavedo (78.54 µg/g) > seeds (9.16 µg/g).

Eriocitrin and hesperidin were the principal flavonoids in albedo (110.29 µg/g and 58.33 µg/g, respectively) and in pulp (353.78 µg/g and 128.58 µg/g, respectively). In fact, flavanones are a typical class of flavonoids in the Citrus genus [1,17]. For instance, Dadwal et al. [20] found that hesperidin was the major phenolic compound in all Citrus medica fractions, including flavedo, albedo, pulp, and seeds, with a high concentration in flavedo (3307.25 µg/g) and lower amounts in seeds (383.02 µg/g). However, these two flavanones were not present in flavedo. Instead, sakuranetin, a naringenin derivate, was the main flavanone in green (30.62 µg/g) and yellow flavedo (47.64 µg/g). Other flavanones like naringin and tangeritin were found in higher concentrations in the citron flavedo (295.15 µg/g and 164.88 µg/g, respectively) [20]. The chromatograms of these flavanones in the pulp are represented in Figure 2. 

Regarding flavanols, rutin was the principal compound in pulp, albedo, flavedo, and seeds, ranging from 3.41 in seeds to 45.99 µg/g FW in green flavedo. The chromatogram of rutin in the pulp is represented in Figure 2. The presence of high amounts of rutin in citron peels was previously reported by Dadwal et al. [20], which were more than 6-fold higher than our results (328.82 µg/g), and by Fratianni et al. [9], with levels 2-fold higher (115.47 µg/g FW). These significant differences could be related to the extraction process, since Dadwal et al. [20] used ultrasound-assisted extraction (UAE) with ethanol at 80% (*v*/*v*) as a solvent, while Fratianni et al. [9] used solid–liquid extraction (SLE) with absolute ethanol as a solvent, a closer approach to our methodology. 

Luteolin, a compound from the flavone class, was only present in small amounts in albedo (0.04 µg/g FW). No other study reported the presence of luteolin. However, another flavone, apigenin, was quantified in mature citron peel by Menichini et al. [17]. More recently, apigenin was not identified in citron peel or pulp [9]. These differences in individual and total phenolic content are related to several factors, including the genotypic diversity of citron fruits, type of soil, and edaphoclimatic conditions during fruit growth (for example, hours of sun exposure, amount of water, humidity of air, and wind) [21]. 

Seeds constitute the citron portion with the lowest amount of flavonoids, where hesperidin and rutin were the main compounds (4.93 and 3.41 µg/g FW, respectively). Other studies comparing citron fractions concluded that seeds were the fraction with the lowest phenolic compound content [20].

Comparing green and yellow flavedo, we could conclude that with the maturity process, there is an increase in flavonoids. However, the content of sakuranetin and quercetin increased, while rutin and quercitrin contents decreased. There was a shift in the major flavonoid, rutin, which was the main compound on green flavedo, while in yellow flavedo the major compound was sakuranetin. Contrarily, Menichini et al. [17] concluded that the green immature fruits had a higher content of flavonoids, where naringin was the major one in the peel (556.0 µg/g FW), while rutin was the major one in pulp (484.7 µg/g FW). The peel of mature yellow fruits presents hesperidin and apigenin but at lower concentrations (9.0 and 58.0 µg/g FW, respectively).

## 3. Materials and Methods

### 3.1. Fruit Samples and Preparation

Citron fruit samples from an orchard located in the north of Portugal (Minho region), were separated into five portions: seeds, pulp, albedo, and flavedo (yellow and green parts of flavedo). Fruits of two different maturation states (5 kg each) were compared, those with green flavedo and those with yellow flavedo (more mature). The five portions were homogenized separately using a grinder homogenizer. Two grams of the homogenized sample were weighted into a 50 mL Falcon tube and combined with 20 mL of 95% ethanol in an Ultra Turrax^®^ T25 (Janke and Kunkel IKA, Stavfen, Germany). The tubes were centrifuged at 2250× *g* for 10 min at 20 °C and the supernatant was isolated to perform the antioxidant capacity assays. For the UHPLC phenolic compounds analysis, two grams of the same homogenizes were mixed with 10 mL of MeOH: ultra-pure H_2_O:formic acid (49.95:49.95:0.10 *v*/*v*/*v*) and sonicated for 10 min. The solution was agitated for 15 min before being centrifuged at 2250× *g* for 10 min at 20 °C. The supernatant was transferred to another Falcon tube, and the extraction was repeated with an additional 10 mL of solvent. The second extract was merged with the first.

### 3.2. Chemicals and Reagents

The phenolic compound standards (4-hydroxybenzoic acid, apigenin, caffeic acid, catechin, chlorogenic acid, ellagic acid, epicatechin, eriocitrin, eriodictyol, gallic acid, gentisic acid, hesperidin, luteolin, naringenin, neochlorogenic acid, *o*-coumaric acid, *p*-coumaric acid, phloridzin, protocatechuic acid, quercetin, quercetin-3-B-D-glucoside, quercitrin, rutin, sakuranetin, sinapic acid, syringic acid, *trans*-ferrulic acid, and vanillic acid), and other reagents (2,2-diphenyl-1-picrylhydrazyl (DPPH), (±)-6-hydroxy-2,5,7,8-tetramethylchromane-2-carboxylic acid (Trolox), Folin-Cioucalteu reagent, sodium carbonate, sodium nitrite, aluminum chloride, sodium hydroxide, β-Carotene, chloroform, Tween^®^ 40, and linoleic acid) were purchased from Sigma-Aldrich (St . Louis, MO, USA). The solvents ethanol and methanol were purchased from Honeywell (Charlotte, NC, USA). Water was purified with a Milli-Q plus system from Millipore (Burlington, MA, USA) with a resistivity of 18.2 MΩ × cm. 

### 3.3. Antioxidant Capacity Assays

#### 3.3.1. β-Carotene Bleaching Assay

The antioxidant capacity of the extracts of each portion was studied in a model system of co-oxidation of the substrates: beta-carotene/linoleic acid, following the method developed by Miller [22]. Accordingly, a 0.2 mg/mL β-carotene in chloroform solution was prepared. After that, 2 mL of the solution was mixed with 20 mg of linoleic acid and 200 mg of Tween^®^ 40 emulsifier. The solvent was then evaporated with the aid of a rotary evaporator. At the same time, some quantity of ultrapure MilliQ^®^ water was oxygenated for around 30 min. Thoroughly mixing 100 mL of this oxygenated water with the evaporated mixture allowed us to prepare the β-carotene emulsion that we used in this assay. An amount of 5 mL of the emulsion was added to 200 μL of each sample and kept at 55 °C in a water bath for 120 min. A control blank sample was read in a spectrophotometer (U-2810, Hitachi, Digilab, Sydney, NSW, Australia) at 470 nm at the beginning of this procedure. Both the samples and the control absorbance were read at the end of this time frame. The Antioxidant Activity Coefficient (AAC) was calculated resorting to the following equation:

(1) $$AAC=\left(\frac{A\;sample-A_{120}\;control}{A_{0}\;control-A_{120}\;control}\right)\times 100$$

where *A*_0_ *control* is the absorbance of the control at the initial time, *A*_120_ *control* is the absorbance of the control after 120 min, and *A sample* is the absorbance of the sample after 120 min in a water bath. All experiments were conducted in triplicate.

#### 3.3.2. DPPH Free Radical Scavenging Assay

This method was carried out following the procedure of Moure et al. [23]. In short, 50 μL of the sample was added to 2 mL of the DPPH radical solution (14.2 g/mL) in a 15 mL Falcon tube and left in the dark for 30 min. The absorbance was measured at 515 nm using a Spectrophotometer (U-2810, Hitachi, Digilab, Sydney, NSW, Australia). A calibration curve (y = 0.8457x − 3.2621, r^2^ = 0.9980) was plotted with Trolox (6-hydroxy-2,5,7,8-tetramethylchroman-2-carboxylic acid) serving as the standard and with concentrations ranging from 5 to 100 g/mL. The results were represented as μg Trolox Equivalents (TE) per gram of fruit. The inhibition percentage was estimated using the following formula:

(2) $$Inhibition\;percentage\;(IP)\left(\%\right)=\left(\frac{A_{control}-A_{sample}}{A_{control}}\right)\times 100$$

where A*_control_* represents the control absorbance and A*_sample_* represents the sample absorbance. All experiments were conducted in triplicate.

### 3.4. Assessment of the Phenolic Characteristics

#### 3.4.1. Total Phenolic Content (TPC) Assessment Assay

To evaluate the phenolic content of the citron extracts, the method described by Singleton et al. [24] was used. Briefly, 1 mL of sample was mixed with 7.5 mL of Folin–Cioucalteu reagent (10% *v*/*v*) and set aside for 5 min, before adding 7.5 mL of Na_2_CO_3_ (60 mg/mL). The absorbance was read after 120 min at 725 nm. A calibration curve (y = 0.0065x − 0.0057, r^2^ = 0.9997) was obtained with concentrations of gallic acid ranging from 5–150 μg/mL. Results were expressed as μg gallic acid equivalents (GAE)/g of fruit. All experiments were conducted using triplicates.

#### 3.4.2. Total Flavonoid Content (TFC) Assessment Assay

In order to estimate the total flavonoid content of the extracts of the citron portions, the method developed by Yoo et al. [25] was applied. In short, 1 mL of the sample extract and 4 mL of ultrapure water were mixed with 0.3 mL of sodium nitrite (50 mg/mL) and vortexed for a few seconds. After 5 min, 0.6 mL of aluminum chloride (100 mg/mL) was added to the mixture and vortexed again. Then, 6 min after that, 2 mL of sodium hydroxide (40 mg/mL) and 2.1 mL of ultrapure water were finally added to the mixture. The absorbance was immediately read at 510 nm. A calibration curve (y = 0.0017x + 0.0165, r^2^ = 0.9986) was analyzed, making use of different concentrations of an epicatechin solution, ranging from 5 to 150 μg/mL. The results are expressed as μg Epicatechin equivalents (EE)/g of fruit. All experiments were conducted in triplicate.

### 3.5. UHPLC-ToF-MS Conditions and Detection of Phenolic Compounds

Detection and quantification of the individual phenolic compounds were executed in a Nexera X2 Shimadzu UHPLC coupled with a 5600+ ToF-MS detector (SCIEX, Foster City, CA, USA) equipped with a Turbo Ion Spray electrospray ionization source operating in positive mode (ESI+). The Acquity UPLC BEH C18 (2.1 mm × 100 mm,1.7 µm) analytical column was utilized in these procedures. The column and auto-sampler were both maintained at 20 °C. The chromatographic separation took place in gradient mode using an aqueous solution of 0.1% formic acid (eluent A) and acetonitrile with 0.1% of formic acid (eluent B) as the mobile phase. The gradient program was applied as follows: 0–0.5 min 90% [A]; 0.5–8 min, decreasing from 90% to 20% [A] and 8–8.1 min 20% [A], completing a total run time of 8.1 min, with the injection volume being 20 μL. Regarding mass spectrometry, the acquisition was carried out in full scan, from 100 to 750 Da using the Analyst^®^ TF 1.7 software (SCIEX, Foster City, CA, USA) and the following parameters: ion source voltage of 5500 V; source temperature of 575 °C; curtain gas (CUR) of 30 psi; Gas 1 and Gas 2 of 55 psi; and declustering potential (DP) of 100 V. To provide accurate mass resolution, the ToF-MS detector was calibrated every six injections in the method’s mass range.

PeakView™ 2.2 and MultiQuant™ 3.0 software (SCIEX, Foster City, CA, USA) were utilized for the identification of phenolic compounds and data processing. The isotope match is automatically presented by the PeakView™ 2.2 software. To identify phenolic compounds, two parameters and their respective equations were used: (1) maximum retention time deviation (ΔRT) of 0.1 min (Equation (3)); (2) exact mass deviation (Δm) with a tolerance of 5 ppm (Equation (4)).

(3) $$\Delta RT=\left(\frac{{RT}_{sample}-{RT}_{standard}}{{RT}_{standard}}\right)\;\times \;100$$

(4) $$\Delta m\;(ppm)=\left(\frac{Exact\;mass-Detected\;Mass}{Exact\;Mass}\right)\;\times \;10^{6}$$

where RT is the retention time.

### 3.6. Statistical Analysis

The statistical analysis of the data was analyzed with IBM^®^ SPSS^®^ Statistics, version 28.0.1.1. (Chicago, IL, USA), employing one-way analysis of variance (ANOVA). Significance was defined at *p* < 0.05. The ANOVA was applied to compare means when the normality of data and the homogeneity of variances were validated, through appropriate statistical tests. The Tukey test was applied to examine the disparities among average values. Results concerning the statistical evaluation are expressed as mean value plus the standard deviation (SD) of three replicates.

## 4. Conclusions

In conclusion, the pulp and peel of *Citrus medica* L. are the fractions of citrus fruits with the highest antioxidant capacity and phenolic compounds. Comparing the antioxidant capacity and individual phenolic compounds, the results indicate that flavonoids were the main phenolic compounds responsible for the antioxidant capacity since the pulp was the fraction with the highest flavonoids content and with the highest antioxidant capacity. Flavanones were the main class of flavonoids in pulp and albedo. In addition, the maturity state influences the composition of phenolic compounds of flavedo, with a decrease in the phenolic acid content and a shift in the major flavonoid from rutin to sakuranetin. 

This study provides valuable information towards the valorization of this ancient fruit concerning its antioxidant properties, highlighting that *Citrus medica* L. has the potential to be used as an excellent source of natural antioxidants. In the food industry, citron can be used in the development of functional foods, promoting Human Health, and delaying food oxidation. In addition, citron imparts citrus flavor, and thus it could be used in the cosmetics and fragrances industries. Other than the antioxidant activity of eriocitrin, hesperidin, sakuranetin, and rutin, these major phenolic compounds of citron demonstrated anti-inflammatory, anti-diabetic, and anticancer activities [26,27,28]; thus, citron can be used in the pharmaceutical industry to prepare new formulations and also food supplements.

The valorization of citron is important to reinforce its status regarding citrus fruit usage so that biodiversity can be maintained, keeping in mind that a decline in it can lead to a problem in the way the ecosystem where it has occurred operates.

## Figures and Tables

**Table 1:** Antioxidant capacity of different fractions of citron (*Citrus medica* L.), including pulp, peel, and seeds, using two methods: DPPH radical scavenging assay and the β-carotene bleaching assay.

Portion of Citron	DPPH Radical Scavenging Assay		AAC
	IP (%)	μg TE/g FW	
Pulp	18.38 ± 0.117 ^e^	254.9 ± 1.384 ^e^	168.2 ± 11.24 ^d^
Albedo	3.808 ± 0.001 ^a^	83.60 ± 0.001 ^a^	37.09 ± 5.619 ^b^
Green Flavedo	7.533 ± 0.117 ^d^	127.6 ± 1.384 ^d^	117.9 ± 3.746 ^c^
Yellow Flavedo	10.51 ± 0.351 ^b^	162.9 ± 4.153 ^c^	111.3 ± 9.366 ^c^
Seeds	5.464 ± 0.117 ^c^	104.2 ± 1.384 ^b^	19.87 ± 7.493 ^a^

The results are expressed as mean ± standard deviation (SD), from three replicates. The superscript letters indicate the statistical analysis. Different letters indicate a significant difference (*p* < 0.05). AAC—Antioxidant Activity Coefficient; TE—Trolox Equivalents; IP—inhibition percentage; FW—Fresh Weight.

**Table 2:** Total phenolic content (TPC) and total flavonoid content (TFC) of different fractions of citron (*Citrus medica* L.), including pulp, peel, and seeds.

Portion of Citron	Total Phenolic Content μg GAE/g FW	Total Flavonoids Content μg EE/g FW
Pulp	564.5 ± 0.001 ^b^	432.9 ± 4.138 ^d^
Albedo	447.1 ± 0.001 ^a^	339.3 ± 4.138 ^c^
Green Flavedo	1010.3 ± 1.093 ^e^	146.1 ± 4.138 ^b^
Yellow Flavedo	1198.8 ± 1.093 ^d^	149.1 ± 8.276 ^b^
Seeds	713.6 ± 1.093 ^c^	26.18 ± 8.276 ^a^

The results are expressed as mean ± standard deviation (SD), from three replicates. The superscript letters indicate the statistical analysis. Different letters indicate a significant difference (*p* < 0.05). GAE—gallic acid equivalents; EE—Epicatechin Equivalents; FW—Fresh Weight.

**Table 3:** Total and individual phenolic acid content (µg/g FW) in different fractions of citron (*Citrus medica* L.), including pulp, peel, and seeds, determined with UHPLC-ToF-MS.

Phenolic Acids	Pulp	Albedo	Green Flavedo	Yellow Flavedo	Seeds	LOD
Chlorogenic acid	n.d.	n.d.	n.d.	5.54 ± 0.07	n.d.	0.5
*o*-coumaric acid	12.01 ± 0.44 ^c^	37.54 ± 0.16 ^e^	23.21 ± 0.38 ^d^	9.52 ± 0.64 ^b^	0.89 ± 0.05 ^a^	0.5
*p*-coumaric acid	1.73 ± 0.01 ^b^	n.d.	8.53 ± 0.02 ^d^	3.90 ± 0.32 ^c^	0.92 ± 0.06 ^a^	0.25
*trans*- ferulic acid	n.d.	n.d.	2.22 ± 0.03 ^b^	1.28 ± 0.04 ^a^	n.d.	1
**∑**	13.74	37.54	33.96	20.24	1.81	

The results are expressed as mean ± standard deviation (SD) from three replicates. The superscript letters indicate the statistical analysis. Different letters indicate a significant difference (*p* < 0.05). The summation (∑) of each class of phenolic compounds is highlighted in bold. LOD—Limit of Detection (expressed as µg/g); n.d.—not detected.

**Table 4:** Total and individual flavonoid content (µg/g FW) in different fractions of citron (*Citrus medica* L.), including pulp, peel, and seeds, determined with UHPLC-ToF-MS.

Flavonoids	Pulp	Albedo	Green Flavedo	Yellow Flavedo	Seeds	LOD
**Flavanones**						
Eriocitrin	353.78 ± 16.98 ^b^	110.29 ± 0.39 ^a^	n.d.	n.d.	n.d.	0.25
Hesperidin	128.58 ± 3.78 ^c^	58.33 ± 2.24 ^b^	n.d.	n.d.	4.93 ± 0.03 ^a^	0.01
Eriodyctiol	n.d.	0.29 ± 0.01	n.d.	n.d.	n.d.	0.025
Sakuranetin	n.d.	0.14 ± 0.01 ^a^	30.62 ± 0.80 ^b^	47.64 ± 0.05 ^c^	0.08 ± 0.02 ^a^	0.05
∑	482.36	169.05	30.62	47.64	5.01	
**Flavanols**						
Rutin	23.42 ± 0.99 ^c^	0.25 ± 0.01 ^a^	45.99 ± 2.02 ^e^	35.92 ± 2.51 ^d^	3.41 ± 0.06 ^b^	0.10
Quercetin	1.12 ± 0.01 ^e^	0.06 ± 0.001 ^b^	0.16 ± 0.001 ^c^	0.32 ± 0.01 ^d^	0.03 ± 0.001 ^a^	0.05
Isoquercetin	0.68 ± 0.05 ^b^	n.d.	1.42 ± 0.02 ^d^	1.29 ± 0.04 ^c^	0.11 ± 0.01 ^a^	0.025
Quercitrin	n.d.	n.d.	0.35 ± 0.02 ^b^	0.12 ± 0.01 ^a^	0.60 ± 0.01 ^c^	0.10
∑	25.22	0.31	47.92	37.65	4.15	
**Flavones**						
Luteolin	n.d.	0.04 ± 0.01	n.d.	n.d.	n.d.	0.01
**∑** **Total Content**	507.58	169.4	78.54	85.29	9.16	

The results are expressed as mean ± standard deviation (SD) from three replicates. The superscript letters indicate the statistical analysis. Different letters indicate a significant difference (*p* < 0.05). The summation (Σ) of each class of phenolic compounds is highlighted in bold. LOD—Limit of Detection (expressed as µg/g); n.d.—not detected.

## Data Availability

Data will be made available on request.

## Supplementary Materials

The following supporting information can be downloaded at: https://www.mdpi.com/article/10.3390/molecules29153533/s1, Table S1: Validation of methodology for determination of 28 phenolic compounds through UHPLC-ToF-MS.
