# Supplementary material for: Unlocking the Potential of Citrus medica L.: Antioxidant Capacity and Phenolic Profile across Peel, Pulp, and Seeds
Source: Molecules. 2024 Jul 27;29(15):3533. doi: 10.3390/molecules29153533 (PMC11313705; doi:10.3390/molecules29153533)
Supplement: Supplementary file 1 [file molecules-29-03533-s001.zip › molecules-3098049-supplementary.pdf]

Supplementary Material:

Table S1. Validation of methodology for determination of 28 phenolic compounds through UHPLC-ToF-MS.

#	Phenolic Compound	rt (min)	Slope	Intercept	R^2	LOQ	Molecular Formula	Molecular Weight (Da)	$[M+H]^+$	Structural subclass
**1**	Gallic acid	1.15	88361.520	-1153.315	0.9937	0.2500	$C_7H_6O_5$	170.022	171.0288	Benzoic acid
**2**	Neochlorogenic acid	1.36	378781.724	-5977.745	0.9957	0.2500	$C_{16}H_{18}O_9$	354.095	355.10236	Hydroxycinnamic acid
**3**	Chlorogenic acid	3.18	976950.083	1573.633	0.9941	0.5000	$C_{16}H_{18}O_9$	354.095	355.10236	Hydroxycinnamic acid
**4**	Catechin	3.23	1062716.913	-11923.080	0.9955	0.1000	$C_{15}H_{14}O_6$	290.079	291.08631	Flavan-3-ols
**5**	4-Hydroxybenzoic acid	3.30	306792.256	8313.451	0.9930	1.0000	$C_7H_6O_3$	138.032	139.03897	Benzoic acid
**6**	Gentisic acid	3.43	102855.556	-3054.630	0.9671	2.5000	$C_7H_6O_4$	154.027	155.03389	Benzoic acid
**7**	Ellagic acid	3.43	103460.000	2165.000	0.9818	2.5000	$C_{14}H_6O_8$	302.197	303.01354	Benzoic acid
**8**	Protocatechiuc acid	3.46	110309.158	-1975.216	0.9923	1.0000	$C_7H_6O_4$	154.027	155.03389	Benzoic acid
**9**	Vanillic acid	3.52	594908.502	13553.265	0.9971	0.2500	$C_8H_8O_4$	168.042	169.04954	Benzoic acid
**10**	Caffeic acid	3.56	572926.467	9247.891	0.9948	0.1000	$C_9H_8O_4$	180.042	181.04954	Hydroxycinnamic acid
**11**	Syringic acid	3.57	711371.934	10602.012	0.9970	0.1000	$C_9H_{10}O_5$	198.053	199.0601	Benzoic acid
**12**	Epicatechin	3.58	2243830.040	-24211.369	0.9971	0.0500	$C_{15}H_{14}O_6$	290.079	291.08631	Flavan-3-ols
**13**	Eriocitrin	4.07	1259869.801	1679.805	0.9992	0.0100	$C_{27}H_{32}O_{15}$	596.174	597.1814	Flavanone
**14**	*p*-coumaric acid	4.12	468533.366	6690.286	0.9973	0.2500	$C_9H_8O_3$	164.047	165.05462	Hydroxycinnamic acid
**15**	Rutin	4.15	1542397.933	-2269.744	0.9939	0.0250	$C_{27}H_{30}O_{16}$	610.153	611.16066	Flavonols
**16**	Sinapic acid	4.25	643118.768	3628.455	0.9973	0.2500	$C_{11}H_{12}O_5$	224.068	225.07575	Hydroxycinnamic acid
**17**	Isoquercetin	4.28	3216557.629	-8864.405	0.9967	0.0250	$C_{21}H_{20}O_{12}$	464.095	465.10275	Flavonols
**18**	*trans*-ferulic acid	4.32	591879.345	4470.335	0.9975	0.2500	$C_{10}H_{10}O_4$	194.058	195.06519	Hydroxycinnamic acid
**19**	Hesperidin	4.61	1821852.193	20280.356	0.9943	0.0100	$C_{28}H_{34}O_{15}$	610.190	611.19705	Flavanone
**20**	Quercitrin	4.63	1058927.972	16609.043	0.9948	0.0250	$C_{21}H_{20}O_{11}$	448.101	449.10784	Flavonols
**21**	*o*-coumaric acid	4.89	324317.154	2960.646	0.9973	0.2500	$C_9H_8O_3$	164.047	165.05462	Hydroxycinnamic acid
**22**	Phloridzin	4.89	112646.365	-331.706	0.9965	0.2500	$C_{21}H_{24}O_{10}$	436.137	437.14422	Dihydrochalcones
**23**	Eriodyctiol	5.50	2758207.800	578.031	0.9972	0.0250	$C_{15}H_{12}O_6$	288.063	289.07066	Flavanone
**24**	Luteolin	5.61	4807419.921	29212.500	0.9979	0.0050	$C_{15}H_{10}O_6$	286.048	287.05501	Flavone
**25**	Quercetin	5.67	4226320.297	38919.900	0.9967	0.0250	$C_{15}H_{10}O_7$	302.043	303.04993	Flavonols
**26**	Naringenin	6.01	4912652.261	12315.809	0.9978	0.0025	$C_{15}H_{12}O_5$	272.068	273.07575	Flavanone
**27**	Apigenin	6.08	6668364.245	45284.725	0.9978	0.0050	$C_{15}H_{10}O_5$	270.053	271.0601	Flavone
**28**	Sakuranetin	7.16	10014124.417	65052.552	0.9980	0.0050	$C_{16}H_{14}O_5$	286.084	287.0914	Flavanone
